# Failure of early non-invasive ventilation in preterm infants with respiratory distress syndrome in current care practice in Spanish level-III neonatal intensive care units – a prospective observational study

**DOI:** 10.3389/fped.2023.1098971

**Published:** 2023-02-21

**Authors:** Hector Boix, Cristina Fernández, María del Mar Serrano Martín, Luis Arruza, Ana Concheiro, Ana Gimeno, Ana Sánchez, Segundo Rite, Francisco Jiménez, Paula Méndez, Juan José Agüera, on behalf of the VENTIS study research group

**Affiliations:** ^1^ Division of Neonatology, Hospital Universitario Dexeus, Barcelona, Spain; ^2^ Department of Neonatology, Hospital Universitario Vall d’Hebron, Barcelona, Spain; ^3^ Division of Neonatology, Regional University Hospital of Malaga, Málaga, Spain; ^4^ Department of Neonatology, Hospital Clínico San Carlos, Instituto de Investigación Sanitaria del Hospital Clínico San Carlos (IdISSC), Madrid, Spain; ^5^ Department of Neonatology, Álvaro Cunqueiro University Hospital, Vigo, Spain; ^6^ Division of Neonatology, University and Polytechnic Hospital La Fe, Valencia, Spain; ^7^ Department of Neonatology, Hospital Universitario La Paz, Madrid, Spain; ^8^ Division of Neonatology, Hospital Universitario Miguel Servet, Zaragoza, Spain; ^9^ Department of Neonatology and Neonatal Intensive Care Unit, Hospital Infantil Universitario Virgen del Rocio, Seville, Spain; ^10^ Division of Paediatrics, Section of Neonatology, Hospital Universitario Puerta del Mar, Cadiz, Spain; ^11^ Department of Paediatrics, Neonatology Section, Virgen de la Arrixaca, University Hospital, Murcia, Spain

**Keywords:** respiratory distress syndrome, non-invasive respiratory ventilation, continuous positive airway pressure, preterm infant, surfactant, less invasive surfactant administration

## Abstract

**Introduction:**

Despite advances in respiratory distress syndrome (RDS) management over the past decade, non-invasive ventilation (NIV) failure is frequent and associated with adverse outcomes. There are insufficient data on the failure of different NIV strategies currently used in clinical practice in preterm infants.

**Methods:**

This was a prospective, multicenter, observational study of very preterm infants [gestational age (GA) <32 weeks] admitted to the neonatal intensive care unit for RDS that required NIV from the first 30 min after birth. The primary outcome was the incidence of NIV failure, defined as the need for mechanical ventilation for <72 h of life. Secondary outcomes were risk factors associated with NIV failure and complication rates.

**Results:**

The study included 173 preterm infants with a median GA of 28 (IQR 27–30) weeks and a median birth weight of 1,100 (IQR 800–1,333) g. The incidence of NIV failure was 15.6%. In the multivariate analysis, lower GA (OR, 0.728; 95% CI, 0.576–0.920) independently increased the risk of NIV failure. Compared to NIV success, NIV failure was associated with higher rates of unfavorable outcomes, including pneumothorax, intraventricular hemorrhage, periventricular leukomalacia, pulmonary hemorrhage, and a combined outcome of moderate-to-severe bronchopulmonary dysplasia or death.

**Conclusion:**

NIV failure occurred in 15.6% of the preterm neonates and was associated with adverse outcomes. The use of LISA and newer NIV modalities most likely accounts for the reduced failure rate. Gestational age remains the best predictor of NIV failure and is more reliable than the fraction of inspired oxygen during the first hour of life.

## Introduction

Neonatal respiratory distress syndrome (RDS) is a disorder that mainly affects premature infants at <34 weeks of gestation. It occurs due to surfactant deficiency in the context of immature lungs, which prevents proper tissue oxygenation. RDS is a common cause of morbidity and mortality in preterm infants, with more severe diseases in smaller and more premature neonates.

The objective of respiratory management of preterm infants with or at risk of RDS is to maximize survival while minimizing potential adverse effects, such as bronchopulmonary dysplasia (BPD). The 2019 European Consensus Guidelines on the management of RDS recommend the early use of continuous positive airway pressure (CPAP) of at least 6 cm H_2_O in all babies at risk of RDS who do not need intubation for delivery room stabilization (1). Early initiation of CPAP potentially reduces the need for mechanical ventilation and surfactant replacement (2, 3) while improving clinical outcomes (2, 4, 5).

Although RDS management has evolved dramatically over the last decade, almost half of the infants who are started on CPAP fail this therapy and are ultimately intubated (6, 7). It should be noted that CPAP failure is associated with an increased risk of death and morbidities, including BPD and pneumothorax (8–10).

Studies have shown that birth weight and male gender are risk factors for CPAP failure (9, 11–13). Furthermore, observational data demonstrate that a fraction of inspired oxygen (FiO_2_) exceeding 0.30 in the first hours after birth in preterm neonates on CPAP is a reasonably good test to predict subsequent CPAP failure (9, 14). However, non-adherence to the recommended FiO_2_ threshold is common in clinical practice (15, 16), which can have negative consequences such as the prolonged need for mechanical ventilation and increased incidence of adverse outcomes.

To mitigate CPAP failure, and in parallel with technological advances in the field of neonatal ventilators, different modes of non-invasive ventilation (NIV) have been introduced, with nasal intermittent positive-pressure ventilation (NIPPV) being the most frequently used alternative. This intervention has been found to have variable success rates in different studies (6, 17, 18). Heated and humidified oxygen delivered by a high-flow nasal cannula (HFNC) has also been studied as the first respiratory support. Still, it was found to be inferior to CPAP in terms of failure rate, and infants randomized to HFNC often need rescue therapy with CPAP to avoid intubation (19).

Although several studies have analyzed the factors associated with CPAP failure in premature infants, there is a lack of sufficient and updated data on the failure of the NIV methods currently used in clinical practice, including HFNC and NIPPV. We designed this observational and prospective study (VENTIS) to evaluate the incidence, predictive factors, and clinical outcomes of NIV failure in preterm infants at risk for RDS admitted to Spanish level-III neonatal intensive care units (NICUs).

## Materials and methods

### Study design and patients

VENTIS was a prospective, multicenter, observational study of preterm infants with RDS initially managed with non-invasive respiratory support. Written informed consent was obtained from the parents or guardians of each patient. The study protocol was reviewed and approved in July 2019 by the Clinical Research Ethics Committee of Vall d'Hebron Hospital, Barcelona, and subsequently by the rest of the Institutional Review Boards of the participating hospitals.

This study was conducted at ten level-III NICUs in Spain between November 2019 and March 2021. Measures were taken according to the distribution of patients across the centers to avoid site effects.

Participants were eligible for the study if they were very preterm newborns [gestational age (GA) <32 weeks] admitted to the NICU for RDS requiring NIV (CPAP, NIPPV, or HFNC) in the first 30 min after birth. The ventilation method and administration of surfactant and/or caffeine citrate were performed according to the local clinical practice. The exclusion criteria were intubation in the delivery room, severe respiratory failure requiring mechanical ventilation from birth, clinical chorioamnionitis, prolonged premature rupture of membranes over 2 weeks, or the presence of a major congenital anomaly.

### Data collection and endpoints

The primary outcome was the incidence of NIV failure, defined as the need for mechanical ventilation within the first 72 h of life. As secondary outcomes, we examined ventilatory outcomes and neonatal morbidities to identify the risk factors associated with NIV failure. The pregnancy and delivery characteristics recorded included antenatal corticosteroid use, mode of delivery, gestational age, sex, birth weight, and Apgar score. Furthermore, we collected data on the type of ventilatory support used, duration and timing of ventilation, ventilator and respiratory parameters, surfactant and/or caffeine administration, FiO_2_ before surfactant administration, and moment of initiation of Kangaroo Mother Care. Clinical outcomes were also recorded, including hospital stay, duration of supplemental oxygen, need for home oxygen, neonatal morbidities/complications, including BPD (need for respiratory support and/or additional oxygen at 36 postmenstrual weeks), air leak syndrome, grade III-IV intraventricular hemorrhage (IVH), periventricular leukomalacia (PVL), retinopathy of prematurity (ROP) >2, patent ductus arteriosus (PDA) with surgical treatment, necrotizing enterocolitis (NEC) Bell stage ≥2, pulmonary hemorrhage, and death.

Data were collected using a case record form developed for this study. The inclusion visit was performed at 24 h of life, and the infants were followed up at 72 h and at hospital transfer, discharge, or death. Information corresponding to the first hour after birth was retrospectively recorded.

### Statistical analysis

Based on the study by Dargaville et al. we estimated that NIV failure would occur in 22% of infants at risk of RDS (9). A sample size of 159 subjects provided an estimate of incidence with a 95% confidence interval (95%CI) with ±7% precision, accounting for 15% invalid data.

Descriptive analysis was performed by calculating frequencies and percentages for categorical variables. The central tendency (mean and median) and dispersion [standard deviation and interquartile range (IQR)] were calculated for quantitative variables. The independent sample *t*-test was used to compare continuous variables, and the *χ*^2^ test or Fisher's exact test was used to compare categorical variables. All analyses are presented for the entire cohort and gestational age.

Multiple logistic regression was used to identify potential predictors of NIV success or failure by calculating odds ratios (OR) with 95% confidence intervals (CI). The variables selected for the multivariate analysis were those corresponding to *p*-values less than 0.1 in the univariate analysis. Statistical significance was set at *p* < 0.05; analyses were performed using the available data without imputation of the missing data. Statistical analyses were performed using the SAS software (version 9.3, SAS Institute Inc., Cary, NC, United States).

## Results

### Patient characteristics

**Figure 1:**
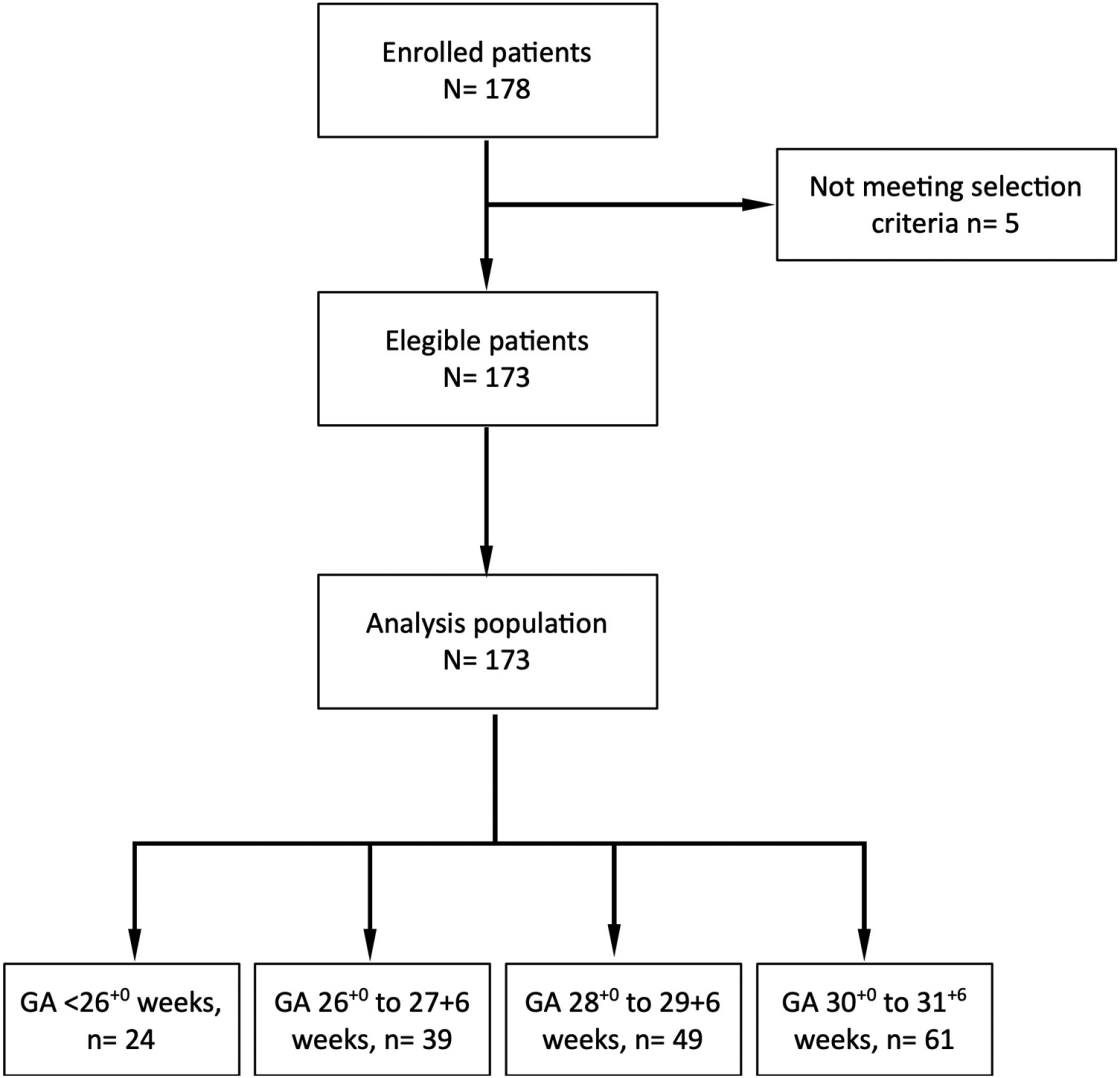
Patient distribution in the VENTIS study. GA, gestational age.

A total of 173 infants were included in the study. Figure 1 shows the flowchart of the study. All infants had complete data and were analyzed.

The characteristics of gestational and newborn infants are shown in Table 1, overall and by GA group. We observed that most pregnancies were singleton (61.8%), and 80.9% of women had received a complete course of antenatal corticosteroids. Cesarean section occurred in 66.6% of the cases. The proportion of male newborns in the study was 50.3%. The median birth weight was 1,100 g (IQR:800–1333).

**Table 1:** Characteristics of the patients included in the VENTIS study by gestational age.

Characteristics	<26+^0/7^ weeks (*n* = 24)	26+^0/7^ to 27+^6/7^ weeks (*n* = 39)	28+^0/7^ to 29+^6/7^ weeks (*n* = 49)	30+^0/7^ to 31+^6/7^ weeks (*n* = 61)	Total population (*n* = 173)
**Mother**					
Multiple pregnancy, *n* (%)	13 (54.2)	13 (33.3)	15 (30.6)	24 (41.0)	66 (38.2)
**Gestation**					
Prenatal corticosteroid use, *n* (%)	24 (100)	35 (89.7)	47 (95.9)	56 (91.8)	162 (93.6)
Complete course	21 (87.5)	27 (77.1)	39 (83.0)	44 (78.6)	131 (80.9)
Incomplete course	3 (12.5)	8 (22.9)	8 (17.0)	12 (21.4)	31 (19.1)
**Mode of delivery, *n* (%)**					
Vaginal	10 (41.7)	15 (38.5)	11 (22.4)	20 (32.8)	56 (32.4)
Caesarean	14 (58.3)	24 (61.5)	38 (77.6)	41 (67.2)	117 (66.6)
**Newborn infant**					
Male sex, *n* (%)	13 (54.2)	15 (38.5)	28 (57.1)	31 (50.8)	87 (50.3)
Gestational age, weeks	−	−	−	−	28.2 (27.0–30.0)
Weight, g	742.5 (650.0–777.5)	900.0 (770.0–1,030.0)	1,128.0 (910.0–1,272.0)	1,440.0 (1,283.0–1,680.0)	1,100.0 (800.0–1,333.0)
Apgar score at 5 min	8.5 (8.0–9.0)	9.0 (8.0–9.0)	9.0 (8.0–9.0)	8.0 (8.0–9.0)	9.0 (8.0–9.0)

If not otherwise indicated, data are the median (IQR, interquartile range).

### Management of respiratory distress

NIV was initiated at a median time of 1.0 min (IQR:0–3) after birth, with CPAP being the most common type of NIV used as the first ventilatory support option (Table 2). Surfactant was administered to 79 infants (45.7%) in the overall study population. Most treated patients (64.6%) received a single dose of surfactant at a median time from the birth of 3 h (IQR:2–6). The surfactant was administered using the less invasive surfactant administration (LISA) method in 88.6% of the infants, the INtubation SURfactant administration and Extubation (INSURE) method in 2.5%, or during mechanical ventilation (MV) in 8.5%. The median FiO_2_ at the time of surfactant administration was 0.35 (IQR:0.30–0.40). Caffeine was administered to 96.5% of the study population.

**Table 2:** Management of respiratory distress syndrome in the VENTIS study by gestational age.

Characteristics	<26+^0/7^ weeks (*n* = 24)	26+^0/7^ to 27+^6/7^ weeks (*n* = 39)	28+^0/7^ to 29+^6/7^ weeks (*n* = 49)	30+^0/7^ to 31+^6/7^ weeks (*n* = 61)	Total population (*n* = 173)
Time from birth to first NIV, minutes	1.0 (0.0–5.0)	1.0 (0.0–1.0)	1.0 (0.0–3.0)	1.0 (0.0–5.0)	1.0 (0.0–3.0)
**Type of initial NIV, *n* (%)**					
CPAP	9 (37.5)	19 (48.7)	28 (57.1)	41 (67.2)	97 (56.1)
NIPPV	15 (62.5)	20 (51.3)	21 (42.9)	20 (32.8)	79 (43.9)
HFNC	0	0	0	0	0
**Type of NIV between 24 and 72 h, *n* (%)**					
CPAP	7 (41.2)	24 (66.7)	30 (66.7)	34 (69.4)	95 (64.6)
NIPPV	10 (58.8)	11 (30.6)	13 (28.9)	7 (14.3)	41 (27.9)
HFNC	0 (0)	1 (2.8)	2 (4.4)	8 (16.3)	11 (7.5)
Surfactant *n* (%)	17 (70.8)	20 (51.3)	26 (53.1)	16 (26.2)	79 (45.7)
LISA/MIST	14 (82.4)	18 (90.0)	23 (88.5)	15 (93.8)	70 (88.6)
INSURE	0 (0.0)	0 (0.0)	2 (7.7)	0 (0.0)	2 (2.5)
SFT+ mechanical ventilation	3 (17.6)	2 (10.0)	1 (3.8)	1 (6.3)	7 (8.9)
FiO_2_ prior to surfactant	0.33 (0.30–0.40)	0.33 (0.30–0.40)	0.38 (0.30–0.40)	0.33 (0.30–0.40)	0.35 (0.30–0.40)
Surfactant dose, mg/kg	200 (184–200)	192 (176–208)	200 (184–200)	192 (176–200)	200 (184–200)
More than one dose of surfactant, *n* (%)	8 (47.1)	10 (50.0)	9 (34.6)	1 (6.3)	28 (35.4)
Time from birth to surfactant, h	4.0 (3.0–8.0)	2.8 (2.0–5.5)	3.0 (2.0–6.0)	6.0 (2.0–6.5)	3.0 (2.0–6.0)
Caffeine, *n* (%)	24 (100.0)	39 (100.0)	49 (100.0)	55 (90.2)	167 (96.5)

CPAP, continuous positive airway pressure; HFNC, high-flow nasal cannula; NIV, non-invasive ventilation; NIPVV, nasal intermittent positive-pressure ventilation; SFT, surfactant. If not otherwise indicated, data are the median (IQR, interquartile range).

### Non-invasive ventilation outcome and risk analysis

**Figure 2:**
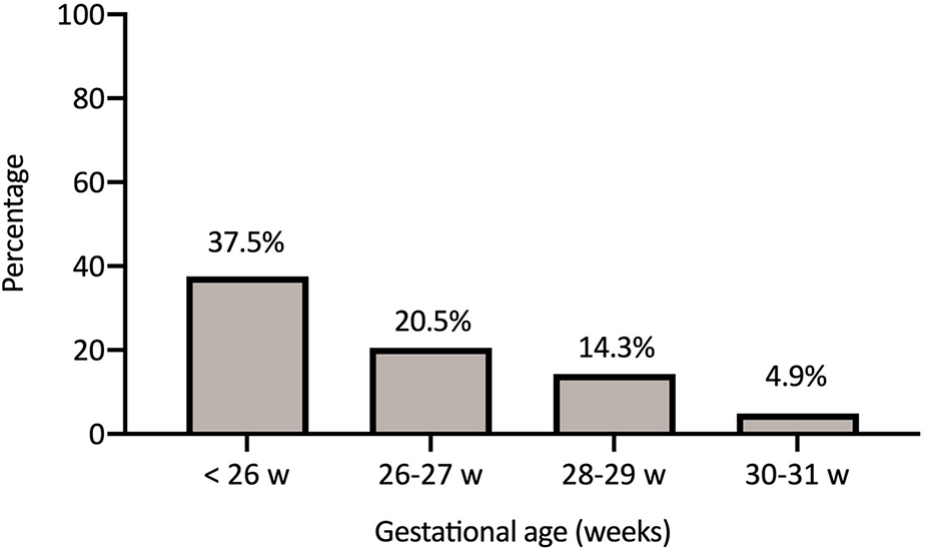
Non-invasive ventilation failure rate according to gestational age.

In general, NIV failure occurred in 27 of 173 infants (15.6%). These infants ultimately required MV within the first 72 h of life. The frequency of NIV failure increased significantly with decreasing gestational age at birth (Figure 2). A total of 124 infants remained on NIV at 72 h of life. The treatment failure rate was 14.6% for CPAP and 17.1% for NIPPV.

To identify variables associated with the outcome of NIV, the success and failure groups of NIV were compared (Table 3). The multivariate regression model confirmed a significant association between GA and NIV failure; the odds of escalation of therapy to MV before 72 h of life increased by 37% at each week of gestation (OR,0.728; 95% CI, 0.576–0.920). Prenatal corticosteroid use (no vs. yes; OR, 7.211; 95% CI, 1.218–42.701) and surfactant administration (no vs. yes; OR,0.054; 95% CI, 0.012–0.252) were also independently associated with NIV failure.

**Table 3:** Comparison of NIV success and NIV failure groups – univariate analysis.

Characteristics	NIV success (*n* = 146)	NIV failure (*n* = 27)	*p-*value
FiO_2_ 1 h	0.30 (0.21–0.40)	0.30 (0.25–0.40)	n.s.
FiO_2_ 1 h–2 h	0.25 (0.21–0.30)	0.27 (0.25–0.31)	0.0261
FiO_2_ 2–6 h	0.21 (0.21–0.29)	0.30 (0.25–0.35)	<0.0001
FiO_2_ 1 h and 1–2h	0.30 (0.21–0.40)	0.31 (0.25–0.45)	n.s.
FiO_2_ 1-, 1–2 h, and 2–6 h	0.30 (0.24–0.40)	0.35 (0.30–0.45)	n.s.
pCO_2_ 1 h	51.5 (46.3–58.2)	51.0 (44.0–56.8)	n.s.
pCO_2_ 1–2 h	51.0 (45.0––57.4)	45.9 (41.5–63.5)	n.s.
pCO_2_ 2–6 h	44.3 (40.0–50.0)	44.2 (41.0–49.0)	n.s.
pCO_2_ 1 and 1–2 h	52.2 (46.3–58.4)	50.5 (44.0–58.0)	n.s.
pCO_2_ 1, 1–2 h, and 2–6 h	52.1 (46.2–58.0)	51.0 (44.0–59.0)	n.s.
**NIV – 1st h of life**			
CPAP/NIPPV, n/n	82/64	15/12	n.s.
CPAP (cmH2O)	5.4 (5.0–6.0)	6.0 (5.0–6.0)	n.s.
MAP in NIPPV (cmH2O)	7.1 (6.8–7.5)	7.4 (5.8–8.9)	n.s.
**NIV – 1st 24 h of life**			
CPAP/NIPPV, n/n	88/58	15/12	n.s.
CPAP (cmH2O)	5.9 (5.0–6.4)	6.0 (6.0–7.5)	0.0136
MAP in NIPPV (cmH2O)	7.4 (6.8–8.0)	7.8 (6.9–8.5)	n.s.
**NIV – between 24 h and 72 h of life**			
CPAP/NIPPV, n/n	92/33	3/8	0.0045
CPAP (cmH2O)	6.0 (5.0–6.3)	7.6 (7.0–8.2)	n.s.
MAP in NIPPV (cmH2O)	7.0 (6.5–7.8)	8.4 (7.5–9.3)	0.0347
**Kangaroo mother care**			
Between 24 h and 48 h of life, n (%)	15 (10.3)	1 (3.7)	n.s.
Between 48 h and 72 h of life, n (%)	29 (19.9)	1 (3.7)	n.s.
**Pregnancy and birth outcomes**			
Sex (male/female), n/n	71/75	16/11	n.s.
Gestational age			0.0003
<26+^0/7^ weeks, *n* (%)	15 (10.3)	9 (33.3)	
26+^0/7^ to 27+^6/7^ weeks, *n* (%)	31 (21.2)	8 (29.6)	
28+^0/7^ to 29+^6/7^ weeks, *n* (%)	42 (28.8)	7 (25.9)	
30+^0/7^ to 31+^6/7^ weeks, *n* (%)	58 (39.7)	3 (11.1)	
Birth weight	1,150 (850–1,400)	900 (745–1,050)	0.0071
Multiple pregnancy/single, n/n	55/91	11/16	n.s.
Mode of delivery (caesarean/vaginal), n/n	95/51	22/5	n.s.
Prenatal corticosteroid use, n (%)	139 (95.2)	23 (85.2)	n.s.
Apgar score at 1 min	7 (6–8)	9 (8–9)	n.s.
Apgar score at 5 min	7 (6–8)	9 (8–9)	n.s.
Surfactant, *n* (%)	54 (37.0)	25 (92.6)	<0.0001
FiO_2_ prior to surfactant	0.35 (0.30–0.40)	0.35 (0.30–0.40)	n.s.
Time from birth to surfactant, h	3.0 (2.0–6.0)	4.0 (3.0–9.5)	n.s.

CPAP, continuous positive airway pressure; HFNC, high-flow nasal cannula; MAP, mean airway pressure; NIPPV, nasal intermittent positive-pressure ventilation; NIV, non-invasive ventilation; n.s., not significant. If not otherwise indicated, data are the median (IQR, interquartile range).

### Ventilatory outcomes and neonatal morbidities

Compared with NIV success, NIV failure was associated with longer hospitalization (median 78 vs. 56 days; *p* = 0.0011), longer duration of supplemental oxygen requirement (median 40 vs. 9 days; *p* = 0.0002), longer duration of NIV (median 37 vs. 16 days; *p* = 0.0126) and need for home oxygen at discharge (14.3% vs. 2.3%, *p* = 0.0337).

There was no difference in the incidence of moderate-to-severe BPD between the groups; however, the need for MV at <72 h of life was associated with significantly higher rates of the combined outcome of moderate-to-severe BPD or death (37% vs. 14.5%; *p* = 0.0114). Nine infants died during the study: two from NEC, one from parenchymal cerebral infarction, one from severe hypoxemic respiratory failure, one from pneumothorax, one from sepsis, and three from septic or cardiogenic shock. The mortality rates did not show statistically significant variation.

In the overall study population, 10.5% of infants had respiratory complications, and 15.7% had common complications of prematurity. Specifically, the failure of non-invasive respiratory support was associated with higher rates of adverse outcomes, including pneumothorax (14.8% vs. 1.4%, *p* = 0.0059), IVH Grade 3 or 4 (14.8% vs. 1.4%, *p* = 0.0059), PVL (11.1% vs. 0.7%, *p* = 0.0125), and pulmonary hemorrhage (14.8% vs. 0%, *p* < 0.0001) (Table 4).

**Table 4:** Clinical outcomes.

Characteristics	Total (*N* = 173)	NIV success (*n* = 146)	NIV failure (*n* = 27)	*p*-value
Length of hospital stay, d	62 (41–79)	56 (41–75)	78 (68–92)	0.0011
Duration of oxygenation, d	16.5 (1.0–45–5)	9 (1–40)	40 (18–72)	0.0002
Duration of NIV, d	20 (5–38)	16 (5–35)	37 (8–55)	0.0126
Duration of MV, d	0.0 (0.0–2.0)	0 (0–0)	6 (2–15)	<0.0001
Discharged on oxygen	6 (3.9)	3 (2.3)	3 (14.3)	0.0337
Death	9 (5.2)	6 (4.1)	3 (11.1)	n.s.
BPD	76 (44.2)	57 (39.3)	19 (70.4)	0.0053
Mild	53 (85.5)	41 (71.9)	12 (63.2)	
Moderate	13 (17.1)	10 (17.5)	3 (15.8)	
Severe	10 (13.2)	6 (10.5)	4 (21.1)	
BPD moderate or severe	23 (23.4)	16 (11)	7 (25.9)	n.s.
BPD mod-severe + death	31 (18.0)	21 (14.5)	10 (37.0)	0.0114
Respiratory complications	18 (10.5)	12 (8.3)	6 (22.2)	0.0413
Pneumothorax	6 (3.5)	2 (1.4)	4 (14.8)	0.0059
Prematurity complications	27 (15.7)	18 (12.4)	9 (33.3)	0.0170
IVH Grade 3 or 4	6 (3.5)	2 (1.4)	4 (14.8)	0.0059
PVL	4 (2.3)	1 (0.7)	3 (11.1)	0.0125
ROP > 2	5 (2.9)	5 (3.4)	0 (0)	n.s.
PDA	4 (2.3)	3 (2.1)	1 (3.7)	n.s.
NEC (bell > 2)	9 (5.2)	8 (5.5)	1 (3.7)	n.s.
Pulmonary haemorrhage	4 (2.3)	0 (0)	4 (14.8)	<0.0001

BPD, bronchopulmonary dysplasia; IVH, Intraventricular haemorrhage; MV, mechanical ventilation NEC, necrotizing enterocolitis; NIV, non-invasive ventilation; PDA, patent ductus arteriosus; PVL, periventricular leukomalacia; ROP, retinopathy of prematurity.

## Discussion

Our observational study included 173 very preterm infants (GA < 32 weeks) who underwent NIV within the first 30 min after birth. The global incidence of NIV failure was 15.6%, and these patients required invasive MV for 72 h. This study allowed us to obtain new data on the global success of NIV in current clinical practice, including the most widely used NIV methods today. In our cohort, 56% of patients underwent CPAP, and 44% had NIPPV in the first 24 h of life. NIPPV was used more often to treat more immature babies and as rescue therapy for those about to fail. Therefore, individual analysis of NIPPV failure could not be performed. European guidelines recommend initiating CPAP of at least 6 cm H_2_O, but the mean pressure with CPAP was significantly lower than with NIPPV on the first hour (5.4 vs. 7.1 cm H_2_O) and this difference persisted throughout. It appears that use of NIPPV enabled clinicians to apply adequate distending pressure.

Previous studies focused only on the CPAP technique and found a higher percentage of failure (failure rates: 20.6%, Rocha et al. (13); 27.8%, Gulczynska et al. (14); 34%, De Jaegere et al. (12); 22%, Dargaville et al. (20). According to our data, if we consider only patients who underwent CPAP in the first 24 h of life, the failure rate was 14.6%. However, we must remember that the aforementioned studies included infants with different GA ranges, the use of LISA or INSURE was not standardized, and the most severe cases of RDS were treated with NIPPV. Dargaville et al. studied a cohort of 25–32 weeks GA patients in a large retrospective study and found a CPAP failure rate of 22%. Given that nearly a decade has passed since the Dargaville study, the higher percentage of success obtained in our cohort can be interpreted as an advance in the use of NIV and surfactant administration techniques or, in general, an improvement in early respiratory management in these patients (20). The Dargaville definition of CPAP failure is the need for mechanical ventilation within 72 h after birth. We used the same definition, but most of our patients received surfactant by a non-invasive technique and were kept on NIV. Therefore, despite using the same definitions, the groups were not comparable.

Given that new NIV methods are being used in addition to CPAP, and its early use has been shown to reduce the use of MV while improving clinical outcomes, it is of utmost importance to know which factors are predictive of NIV failure. Early identification of those premature infants who most likely fail NIV can allow specific therapeutic interventions to be aimed at them. The final multivariate model identified GA as an independent predictor of NIV failure. The risk of unfavorable outcomes increased by 37% with each week of gestational age. Gestational age has been associated with CPAP failure. Gulczyńska et al. found that each gestational week reduced the odds of CPAP failure by 19% in infants with <30 GA (19). Dargaville et al. found a significant association in the most premature infants, 25–28 weeks of GA, in whom each additional week reduced the risk of failure by 39% (9). However, in both cases and unlike our study, GA did not prove to be an independent predictor in multivariate logistic regression.

Interestingly, FiO_2_ between the second and sixth hours of life was predictive of failure in our univariate model but was not significant in the multivariable model. Several authors have pointed out FiO_2_ as a powerful predictor of CPAP failure (21). Specifically, Dargaville et al. found that a FiO_2_ > 0.3 in the first hours of life was predictive of CPAP failure, and this threshold is used in current European RDS guidelines for early rescue administration of surfactants (1, 9). Due to the observational nature of the present study, the surfactant was administered following clinical practice, with a median FiO_2_ slightly higher than that recommended in the European guidelines (1). Therefore, the statistically significant association between surfactant use and NIV failure should be interpreted with caution. This association probably exists because the VENTIS is an observational study and was not designed to evaluate the use of surfactants. All patients with mild RDS who did not receive surfactant were in the NIV success group, and all patients who failed NIV received surfactant at the time of intubation. Furthermore, the correct timing of surfactant use may have contributed to a higher percentage of success of non-invasive techniques. Therefore, proper adherence to the recommendations of the current European RDS guidelines can be confirmed. Early administration of surfactants, especially with the LISA technique using a thin catheter while continuing CPAP, reduces the need for subsequent MV (22–26).

Infants for whom NIV failed were at a considerably higher risk of adverse outcomes and morbidity than those successfully managed with NIV (9, 14, 20). Non-invasive ventilation failure was associated with a higher percentage of BPD and the composite outcome of moderate-to-severe BPD plus death. However, the number of deaths did not differ significantly between the success and failure groups. Furthermore, non-invasive respiratory support failure was associated with higher rates of other unfavorable outcomes, including pneumothorax, IVH, PVL, and pulmonary hemorrhage. All these complications have previously been related to RDS and prematurity, especially in seriously ill infants who need MV because non-invasive support fails. As in the Dargaville study, the duration of hospital stay and oxygen therapy was significantly higher in infants who failed NIV. We also observed that the need for home oxygen at discharge and duration of NIV were significantly higher in the failure group than in the success group. Our data show that the predictive variables for NIV failure evolve towards GA rather than FiO_2_ in the initial hours if the current threshold recommendations for surfactant administration are followed. There is a need to improve respiratory management in premature infants to avoid unfavorable outcomes.

Our study had some limitations. This was an observational study, and the results obtained showed the variability of real-life clinical practice. Although we recorded the PEEP and MAP (median and IQR) for CPAP and NIPPV at intervals (first hour of life, first 24 h of life, 24–72 h of life), we did not record the level of support immediately before failure.

## Conclusion

The incidence of NIV failure in our cohort of very preterm infants managed according to current RDS respiratory management standards was 15.6%. The lower failure rate observed compared with previous studies is most likely due to LISA and the newer NIV modes currently used in our units. The use of NIPPV likely facilitated the use of higher distending pressure. Gestational age remains the best predictor of NIV failure as primary respiratory support in premature infants. FiO_2_ during the first hours of life was not an independent risk factor for NIV failure in our cohort, confirming that FiO_2 _> 0.3 is a good indicator for surfactant administration as recommended in the European guidelines for the management of RDS.

## Data Availability

The raw data supporting the conclusions of this article will be made available by the authors, without undue reservation.
